# Key Role of the CD56^low^CD16^low^ Natural Killer Cell Subset in the Recognition and Killing of Multiple Myeloma Cells

**DOI:** 10.3390/cancers10120473

**Published:** 2018-11-29

**Authors:** Elisabetta Vulpis, Helena Stabile, Alessandra Soriani, Cinzia Fionda, Maria Teresa Petrucci, Elena Mariggio’, Maria Rosaria Ricciardi, Marco Cippitelli, Angela Gismondi, Angela Santoni, Alessandra Zingoni

**Affiliations:** 1 Department of Molecular Medicine, Istituto Pasteur-Fondazione Cenci Bolognetti, Sapienza University of Rome, 00161 Rome, Italy; elisabetta.vulpis@uniroma1.it (E.V.); helena.stabile@uniroma1.it (H.S.); alessandra.soriani@uniroma1.it (A.S.); cinzia.fionda@uniroma1.it (C.F.); marco.cippitelli@uniroma1.it (M.C.), angela.gismondi@uniroma1.it (A.G.); 2 Division of Hematology, Department of Cellular Biotechnologies and Hematology, Sapienza University of Rome, 00161 Rome, Italy; petrucci@bce.uniroma1.it (M.T.P.); mariggio@bce.uniroma1.it (E.M.); 3 Division of Hematology, Department of Clinical and Molecular Medicine, Sapienza University of Rome, 00161 Rome, Italy; ricciardi@bce.uniroma1.it; 4 IRCCS, Neuromed, 86077 Pozzilli, Italy

**Keywords:** natural killer cells, multiple myeloma, immunotherapy, HSCT

## Abstract

Natural Killer (NK) cells play a pivotal role in the immunosurveillance of Multiple Myeloma (MM), but it is still undefined whether the NK cell functional properties underlying their protective activity against MM are confined to distinct NK cell populations. Interestingly, herein we report that the CD56^low^CD16^low^ NK cell subset displayed higher cytolytic activity compared to the other NK cell subsets (i.e., CD56^high^CD16^+/−^, CD56^low^CD16^high^) against MM cells and its activity was impaired in MM patients. Decreased DNAM-1 expression levels were observed on the CD56^low^CD16^low^ NK cells during MM progression. Evaluating NK cell subset frequency after autologous hematopoietic stem cell transplantation, we found that CD56^low^CD16^low^ NK cells recovered earlier after transplantation. Overall, our data denote a key role of CD56^low^CD16^low^ subpopulation in the killing of MM cells and suggest that the reconstitution of CD56^low^CD16^low^ subpopulation after HSCT could be a useful approach of adoptive immunotherapy in the treatment of relapsed/refractory MM patients.

## 1. Introduction

Natural Killer (NK) cells are innate immune effector lymphocytes with a pivotal role in the immune response against cancer cells. NK cell killing of cancer cells depends on the integration of intracellular signaling cascades initiated by the engagement of different cell-surface inhibitory and activating receptors [1]. Multiple myeloma (MM) is a clonal B cell malignancy characterized by expansion of plasma cells (PCs) in the bone marrow (BM) [2]. At present, it is still an incurable disease with a median survival not exceeding five years, and its prognosis has been recently meliorated by the use of autologous hematopoietic stem cell transplantation (HSCT) and by new immunochemotherapeutic approaches [3,4]. Among immune cells that play a role in the surveillance of MM, NK cells have been considered important since they are able to recognize and kill tumor cells. In this regard, among the activating receptors expressed by NK cells, NKG2D, DNAM-1 (CD226) and NKp30 are emerging as key receptors for the recognition of MM cells, being engaged by their ligands expressed by tumor cells [5,6,7] and thus triggering NK cell cytotoxicity [5,7,8]. Due to their anti-myeloma properties, recent interest in the use of NK cells in seeking novel immunotherapeutic approaches for this malignancy has emerged [9,10,11,12]. To this regard, the usage of anti-SLAMF7/CD319 monoclonal antibody elotuzumab exerts its anti-MM activity mainly via NK cell-mediated antibody dependent cellular cytotoxicity (ADCC) through the CD16 receptor and the triggering of CD319/SLAMF7 on NK cells [13,14]. However, it is still undefined whether the functional properties underlying NK cell protective activity against MM are confined to distinct NK cell populations. In this regard, in humans, two major NK cell subsets have been described based on the cell surface density of the low-affinity Fc-receptor γ IIIA (CD16) involved in the NK cell-mediated antibody-dependent cellular cytotoxicity (ADCC), and the neural cell adhesion molecule (NCAM, CD56); CD56^low^CD16^high^ cells represent 90% of circulating peripheral blood (PB) NK cells and are able to mediate natural cytotoxicity and ADCC while, CD56^high^CD16^+/−^ NK cells constituting 10% of PB NK cells and are the main cytokine producers. Although it is still debated whether these subsets represent terminally differentiated NK cells or NK cells at a different stage of maturation, several evidences show that CD56^high^ NK cells represent a more immature stage of differentiation able to generate CD56^low^CD16^high^ in in vitro experiments and humanized mouse models [15,16,17].

Recently, a subset of NK cells with low expression levels of both CD56 and CD16 (CD56^low^CD16^low^) has been described in the BM and PB of pediatric healthy donors and leukemic transplanted patients [18,19,20]. This subset is more abundant in BM with respect to PB and even if, according to the receptor surface phenotype, could represent an intermediate stage of differentiation between CD56^high^CD16^+/−^ and CD56^low^CD16^high^, CD56^low^CD16^low^ NK cells are a multifunctional subset endowed with a potent cytotoxic ability against human HLA class-I-deficient K562 erytroleukemia target cells and leukemia blasts and with a higher ability to produce IFNγ [18,19,21]. Herein we analyzed during MM progression: (i) the distribution of distinct NK cell subsets (i.e., CD56^high^CD16^+/−^, CD56^low^CD16^low^ and CD56^low^CD16^high^) in the BM and PB from MM patients at different disease states and the expression levels of NKG2D, DNAM-1 and NKp30 on these populations; (ii) the functional capability of these distinct NK cell subsets to recognize and kill MM cells and their activity in the course of MM progression; (iii) the distribution and the functionality of these three subsets after autologous HSCT.

## 2. Results and Discussion

### 2.1. Patient Characteristics

A cohort of 72 MM patients at different states of the disease with age ranged between 41 and 87 years, and a percentage of malignant plasma cells (PCs) between 4% and 67% were enrolled. In this cohort of patients, 19 displayed a monoclonal gammopathy of undetermined significance (MGUS), 18 were classified as Smoldering, and 35 had symptomatic MM (18 at onset and 17 at relapse) (Table 1 and Table S1).

### 2.2. CD56^low^CD16^low^ NK Cell Subset Is Enriched in BM from MM Patients

**Figure 1:**
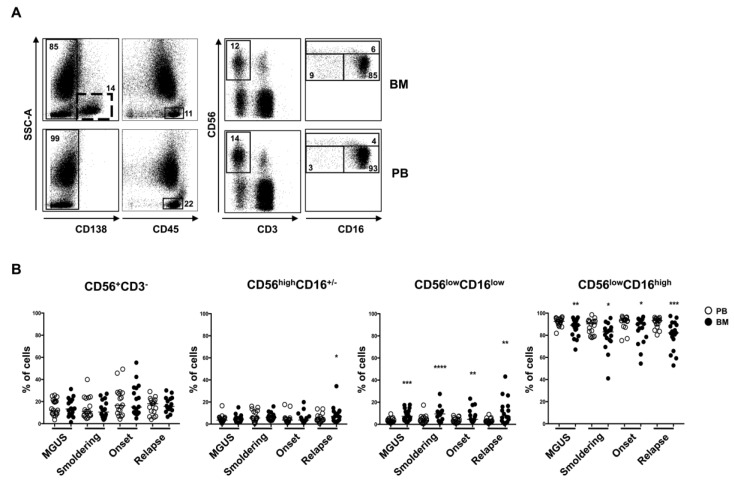
Natural Killer (NK) cell subset distribution in bone marrow (BM) and peripheral blood (PB) of multiple myeloma (MM) patients at different states of the disease. Representative dot plots of NK cells derived from PB and BM of MM patient after CD138^+^ cell gate exclusion, by gating on CD45^+^CD56^+^CD3^−^ cells were shown. NK cell subsets were analyzed considering the expression levels of CD56 and CD16 (**A**). Percentage of total NK cells and NK cell subsets in PB (white circle) and BM (black circle) of MM patients at different states of the disease was shown (**B**) (MGUS, *n* = 19; Smoldering, *n* = 18; Onset, *n* = 18; Relapse, *n* = 17) **** *p* < 0.0001; *** *p* < 0.001; ** *p* < 0.01; * *p* < 0.05. The most significant differences in the NK cell distribution between PB e BM were mainly observed for the CD56^low^ CD16^low^ subset in the monoclonal gammopathy of undetermined significance (MGUS) and Smoldering states.

We first analyzed the distribution of the three distinct NK cell subsets: CD56^high^CD16^+/−^, CD56^low^CD16^low^ and CD56^low^CD16^high^ in the BM and PB of MM patients (Figure 1A). Our findings show no significant differences between total NK cells from the BM and PB samples of MM patients at different disease stages. However, we found a significant reduction of the CD56^low^CD16^high^ NK cell subset accompanied by a corresponding increase of the CD56^low^CD16^low^ NK cells in the BM when compared to PB in all MM states (Figure 1B). The higher expression level of CXCR4 chemokine receptor shown by CD56^low^CD16^low^ NK cell subset may account for their preferential retention in the BM [21]. In addition, we have previously shown that CXCR4 expression does not change on NK cells from MM patients at different disease stages, even though its ligand, the stromal cell derived factor-1 (SDF-1), decreased during MM progression [22]. Furthermore, assuming that the distinct NK cell subsets (i.e., CD56^low^CD16^low^, CD56^high^CD16^+/−^, CD56^low^CD16^high^) represent different maturation stages of NK cells, it is conceivable to hypothesize that higher frequency of the CD56^low^CD16^low^ NK cells in the BM paralleled by increased frequency of CD56^low^CD16^high^ NK cells in the PB is the result of an impaired NK cell differentiation in the tumor microenvironment, as previously observed in patients affected by acute myeloid leukemia (AML) [19]. We also found a different distribution of CD56^high^CD16^+/−^ NK cell subset between BM and PB in relapsed patients with a significant increase in the BM (Figure 1B). Interestingly, we have previously shown that relapsed patients showed the highest level of soluble IL-15 in vivo [23]. This cytokine is important in the BM tumor microenvironment by exerting opposite effects: Promoting an autocrine loop for myeloma cell survival and sustaining NK cell proliferation through its direct or exosome-mediated *trans*-presentation [23]. The ability of IL-15 to preferentially promote the expansion of CD56^high^CD16^+/−^ NK subset may in part explain the increased percentage of this subset in the BM of relapsed patients [24].

### 2.3. BM CD56^low^CD16^low^ NK Cells from MM Patients Show a Decreased Expression of DNAM-1 and NKp30

**Figure 2:**
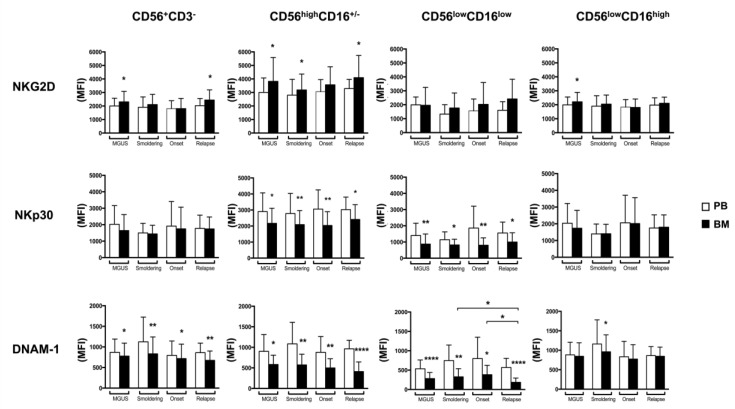
BM and PB NK cell subsets receptor profile of MM patients during disease progression. FACS analysis of surface expression of NKG2D (**A**)**,** NKp30 (**B**) and DNAM-1 (**C**) on total NK cells and NK cell subsets in PB (white histograms) and BM (black histograms) of MM patients at different state disease (MGUS, *n* = 19; Smoldering, *n* = 18; Onset, *n* = 18; Relapse, *n* = 17) was shown. Values are expressed as mean of mean fluorescence intensity (MFI) and error bars represent SD. **** *p* < 0.0001; ** *p* < 0.01; * *p* < 0.05.

To phenotypically characterize both total NK cells and NK cell subsets in MM patients, we evaluated the expression levels of three different activating NK cell receptors namely NKG2D, DNAM-1 (CD226) and NKp30 involved in the recognition and killing of MM. Interestingly, we observed a significant decrease in NKp30 expression levels on BM CD56^high^CD16^+/−^ and CD56^low^CD16^low^ NK cell subsets at all stages of the disease and also a considerable lower expression of this receptor on the CD56^low^CD16^low^ subpopulation (Figure 2). Similarly, a reduction of NKp30 was reported in the context of other haematological malignancies [25,26] and was ascribed to the presence of TGF-β, a cytokine known to downregulate NKp30 expression [27]. In regard to DNAM-1, similarly to NKp30, we observed a significant reduction of the expression of this receptor on BM CD56^high^CD16^+/−^ and CD56^low^CD16^low^ NK cell subsets at all the disease states and also a considerable lower expression of this receptor was detected on CD56^low^CD16^low^ subpopulation (Figure 2). Interestingly, we also noticed a significant decrease of DNAM-1 expression levels during MM progression only on the CD56^low^CD16^low^ NK cell subset (Figure 2). A recent report has shown that in a mouse model, DNAM-1 played an important role in the surveillance of MM and was required for optimal response to different chemotherapeutic agents namely bortezomib and cyclophosphamide [28]. In line with these observations, the expression of DNAM-1 ligands, CD155 and CD112, detected on human primary malignant PCs and MM cell lines [5,7] were upregulated in response to bortezomib and other drugs [5,9,10,29,30]. It should be taken into consideration that the reduced DNAM-1 expression levels during MM progression could be dependent on the presence of its ligands on cancer cells [31,32] and might be associated with an impairment of NK cell-mediated immunosurveillance, as previously observed in myelodysplastic syndrome [33]. Interestingly, beyond MM cells, the DNAM-1/CD155 axis has been also reported to play a key role in the NK cell dependent killing of other haematological malignancies, including acute myeloid leukemic cells [34]. Another important consideration relies on the fact that DNAM-1 expression has been described to be associated with NK cell maturation, being expressed at lower levels on the most immature cells and tumor microenvironment could substantially affect this process [18]. In relation to NKG2D, its levels were almost similar on NK cells derived from BM and PB in all the disease states and as shown in Figure 2, a very heterogeneous expression of this receptor, especially on CD56^low^CD16^low^, CD56^high^CD16^+/−^ NK cells, was found. Previously, Fauriat and co-workers have shown lower but very variable levels of NKG2D expression on PB NK cells from MM patients, when compared to healthy donors [35], while in another study a preferential reduction of NKG2D was observed only on BM NK cells [36]. These discrepancies could be related to the different methodologies and techniques used to identify the cells and to the fact that NKG2D expression is largely modulated by a plethora of factors, including both cytokines [37,38,39] and soluble ligands [40,41].

### 2.4. CD56^low^CD16^low^ NK Cell Subset Is the Major Cytolytic Population Against MM Cells and Is Impaired in MM Patients

**Figure 3:**
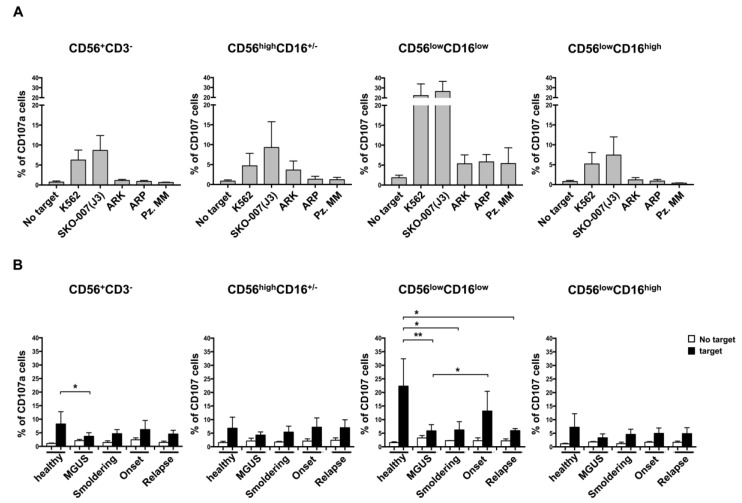
NK cell subset degranulation in MM patients. PBMCs derived from healthy donors were incubated for two hours with distinct targets as indicated, the K562 cell line, three MM cell lines (i.e., SKO-00(J3), ARP and ARK) and primary malignant plasma cells (PCs). Cells were harvested and stained with anti-CD3, anti-CD56, anti-CD16 and anti-CD107 antibodies. Values corresponded to the mean +/−SD of the percentage of CD107^+^ cells of at least three experiments are shown (**A**). Degranulation assay performed as described in panel A using PBMCs derived from patients at different state disease as indicated. Values are expressed as mean percentage of CD107^+^ cells and error bars represent SD (**B**). ** *p* < 0.01; * *p* < 0.05. Healthy donors, *n* = 6; MGUS, *n* = 6; Smoldering, *n* = 4; Onset, *n* = 5; Relapse, *n* = 4).

Next, since it has been described that the CD56^low^CD16^low^ NK cell subset represents the major cytotoxic NK cell population against human HLA class-I-deficient K562 target or acute leukemia blast cells [18], we further investigated the capability of these cells to recognize and kill MM cells. To this aim, three different MM cell lines, SKO-007(J3), ARK and ARP, and primary malignant PCs were used as targets in a degranulation assay. As shown in Figure 3A, the CD56^low^CD16^low^ NK cell subset was endowed with the higher capability to kill not only the highly sensitive K562 cell line but also MM cells even though at different extent, thus strongly suggesting that this subset plays a key role in the recognition and killing of MM cells (Figure 3A and Figure S1). In order to evaluate whether the CD56^low^CD16^low^ NK cell subset functions could be affected in MM patients, we performed a degranulation assay using PB cells isolated from healthy donors or MM patients at different disease states. As shown in Figure 3B, we observed a general impairment of NK cell degranulation in MM patients when compared to total NK cells derived from the healthy donors. Interestingly, the CD56^low^CD16^low^ NK cell subset showed a significant reduction of its degranulation capability at distinct disease states, including MGUS (Figure 3B). Similar levels of NK cell degranulation were also observed in BM NK cells from MGUS and MM patients (Figures S2 and S3).

### 2.5. CD56^low^CD16^low^ NK Cells Expanded Early After Autologous HSCT in MM Patients

**Figure 4:**
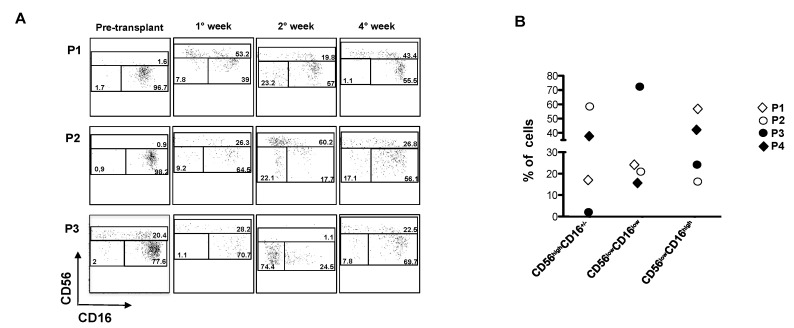
NK cell subset distribution after autologous HSCT. Analysis of NK cell subsets derived from PB of MM patients after transplant. Representative dot plots of NK cell subset distribution on CD45^+^CD56^+^CD3^-^ cells derived from three patients (P1, P2 and P3) at different time after transplant were shown (**A**). Percentage of CD56^high^CD16^+/−^, CD56^low^CD16^low^ and CD56^low^CD16^high^ of four different MM patients (P1, P2, P3 and P4) after two weeks from transplantation. Each symbol represents a patient (**B**).

High-dose melphalan followed by autologous hematopoietic stem cell transplantation (HSCT) represents a common therapeutic approach in relapsed/refractory MM patients to prolong progression free survival [42]. Thus, we further investigated the recovery of NK cell subsets and their functional capability after autologous HSCT in MM patients. NK cell subsets were analysed at different times upon transplantation. Our findings show that the frequency of the CD56^low^CD16^low^ NK cell subset reaches a peak starting from the 2^nd^ week from the transplant and returned to basal levels after four weeks (Figure 4A,B). On the other side, CD56^high^CD16^low^ cells expanded earlier and their percentage remained considerably high after four weeks from the transplant in accordance with previous studies [43]. In regard to the CD56^low^CD16^high^ NK cells, their percentage was still below the basal values after four weeks, suggesting that this subset takes more time to develop. In terms of functionality, a general impairment of NK cell degranulation was observed after four weeks from the transplant [44] and as previously reported [45]. Several studies have shown that despite improved outcomes, relapse after autologous HSCT is frequent in the majority of MM patients [42,46]. Killer immunoglobulin-like receptor (KIR)-ligand mismatched NK cells are determinant in achieving durable remission after haplo-HSCT for acute myeloid leukaemia (AML), exerting a potent anti-leukemia effect [47]. Interestingly, the CD56^low^CD16^low^ NK cell subset has the most rapid and abundant recovery in terms of functional activity with respect to the other NK cell subsets after haplo-HSCT in leukemia patients. Indeed, these cells exhibit the highest percentage of CD107^+^ cells [19] and might strongly contribute to the graft versus leukemia (GVL) effect. Emerging evidence has shown that infusion of allogeneic KIR-mismatched NK cells should be considered in relapsed MM patients with high-risk myeloma and in those relapsing after novel agent-based therapies or early after an autologous HSCT [48,49]. In view of the fact that CD56^low^CD16^low^ NK cells showed the higher cytolytic activity against MM cells, our data strongly suggest that the reconstitution of CD56^low^CD16^low^ subpopulation after HSCT could be a useful approach of adoptive immunotherapy especially in the treatment of relapsed MM patients.

## 3. Materials and Methods

### 3.1. Clinical Samples

PBMCs and BM samples derived from MM patients enrolled at the Division of Hematology (“Sapienza” University of Rome) and control PBMCs were obtained from age-matched healthy donors. All MM patients were classified according to the disease state (Table 1 and Table S1). The BM aspirates and the peripheral blood samples were processed as previously described [5,11]. In some experiments, primary malignant plasma cells were purified from BM aspirates using anti-CD138 magnetic beads (Miltenyi Biotec, Auburn, CA, USA) and more than 95% of the purified cells were CD138^+^CD38^+^. In regard to patients undergoing autologous HSCT, they received a high-dose of melphalan (MEL) before HSCT infusion.

### 3.2. Cell Lines

The human MM cell lines SKO-007(J3), ARK and ARP were provided by P.Trivedi (“Sapienza” University of Rome). The MM cell lines and the human chronic myeloid leukemia cell line K562 were maintained at 37 °C and 5% CO_2_ in RPMI 1640 (Life Technologies, Gaithersburg, MD, USA) supplemented with 10% FCS. All cell lines were mycoplasma free (EZ-PCR Mycoplasma test kit; Biological Industries, Cromwell, CT, USA).

### 3.3. Ethics Statement

Informed and written consent in accordance with the Declaration of Helsinki was obtained from all patients, and approval was obtained from the Ethics Committee of the Sapienza University of Rome (Rif.3373/250914).

### 3.4. Immunofluorescence and FACS Analysis

Analysis of activating receptors on NK cells were performed on PB and BM samples using a gating strategy on CD138^-^CD45^+^CD56^+^CD3^-^CD16^+/−^ cell after CD138^+^ cell (corresponding to PCs) gate exclusion (Figure 1A). The samples were stained with anti-CD138/FITC, anti-CD3/APC-H7, anti-CD56/PE, anti-CD45/PE-Cy7, anti-CD16/PerCP, anti-NKG2D/APC, anti-DNAM-1/FITC (BD Biosciences, San Jose, CA, USA) and anti-NKp30/APC (BioLegend, San Diego, CA, USA) for 25 min at 4 °C [50]. Expression levels of NK cell receptors on NK cell subsets derived from healthy PB donors are shown in Figure S4.

All the samples were acquired using a FACSCanto II (BD Biosciences, San Jose, CA) and data analysis was performed using the FlowJo 9.3.2 program (TreeStar, Ashland, OR, USA).

### 3.5. Ex-vivo Degranulation Assay

As the source of effector cells, we used both PBMCs and BM samples derived from MM patients at different state disease and PBMCs from healthy donors. K562 were used as target cells that were co-incubated with effector cells in complete medium at 2.5:1 effector/target (E/T) ratio for 2 h at 37 °C and 5% CO_2_ [11]. In some experiments, different human cell lines such as SKO-007(J3), ARK and ARP and primary malignant plasma cells were used as targets. Thereafter, cells were washed with PBS/2% FCS and stained with the lysosomal marker CD107a/APC (BD Biosciences, San Jose, CA) and anti-CD3/APC-H7, anti-CD56/PE, anti-CD45/PE-Cy7, anti-CD16/PerCP for 45 min at 4 °C. All the samples were acquired using a FACSCanto II (BD Biosciences, San Jose, CA) and data analysis was performed using the FlowJo 9.3.2 program (TreeStar, Ashland, OR, USA).

### 3.6. Statistical Analysis

In all the experiments, statistic was performed using the unpaired Student t-test to compare the different states of MM, while paired Student *t*-test were used to compare PB and BM, * < 0.05; ** < 0.01; *** < 0.001; **** < 0.0001. Statistical analyses were performed using PRISM 7.0a (GraphPad, La Jolla, CA, USA).

## 4. Conclusions

Recent interest in the use of NK cells for novel immunotherapeutic approaches for multiple myeloma has emerged. However, it is still undefined whether the functional properties underlying NK cell protective activity against MM are confined to distinct NK cell populations. Our findings denote a key role of CD56^low^CD16^low^ subpopulation in the killing of MM cells and suggest that the reconstitution of CD56^low^CD16^low^ subpopulation after HSCT could be a useful approach of adoptive immunotherapy in the treatment of relapsed/refractory MM patients. The CD56^low^CD16^low^ NK cells can be isolated and ex vivo expanded to promote their functional activity in order to ameliorate the outcome of transplants.

## Figures and Tables

**Table 1:** Clinical characteristics of the studied MM patient.

State Disease	Gender		Age			Tumor Burden (% PCs)		
			Range	Mean	SD	Range	Mean	SD
MGUS	Male	12	41–79	63.37	11.54	5–52	5.22	3.61
	Female	7						
Smoldering	Male	4	49–87	70.61	10.05	4–60	19.89	13.97
	Female	14						
Onset	Male	8	41–79	63.35	11.11	6–67	31.56	18.53
	Female	10						
Relapse	Male	9	55–84	70.06	8.47	5–52	32.7	17.51
	Female	8						

PCs: plasma cells; MGUS: monoclonal gammopathy of undetermined significance.

## Supplementary Materials

The following are available online at http://www.mdpi.com/2072-6694/10/12/473/s1; Figure S1. PB derived cells from two representative healthy donors were incubated for two hours without target or with distinct target cells including K562 and various MM cell lines (SKO-007(J3), ARK, ARP) and primary plasma cells (pz MM) as indicated; Figure S2. PB and BM derived cells from the same representative patient were incubated for two hours with or without the target cell line, K562; Figure S3. PB and BM derived cells were incubated for two hours with or without the target cell line, K562; Figure S4. Expression levels of NK cell receptors on NK cell subsets derived from healthy PB donors. Table S1. Therapeutic history of relapsed patients.
