# Supplementary material for: Key Role of the CD56lowCD16low Natural Killer Cell Subset in the Recognition and Killing of Multiple Myeloma Cells
Source: Cancers (Basel). 2018 Nov 29;10(12):473. doi: 10.3390/cancers10120473 (PMC6317053; doi:10.3390/cancers10120473)
Supplement: Supplementary file 1 [file cancers-10-00473-s001.pdf]

Supplementary Materials: Key role of the CD56^{low}CD16^{low} Natural Killer cell subset in the recognition and killing of Multiple Myeloma cells

Elisabetta Vulpis, Helena Stabile, Alessandra Soriani, Cinzia Fionda, Maria Teresa Petrucci, Elena Mariggio', Maria Rosaria Ricciardi, Marco Cippitelli, Angela Gismondi, Angela Santoni and Alessandra Zingoni

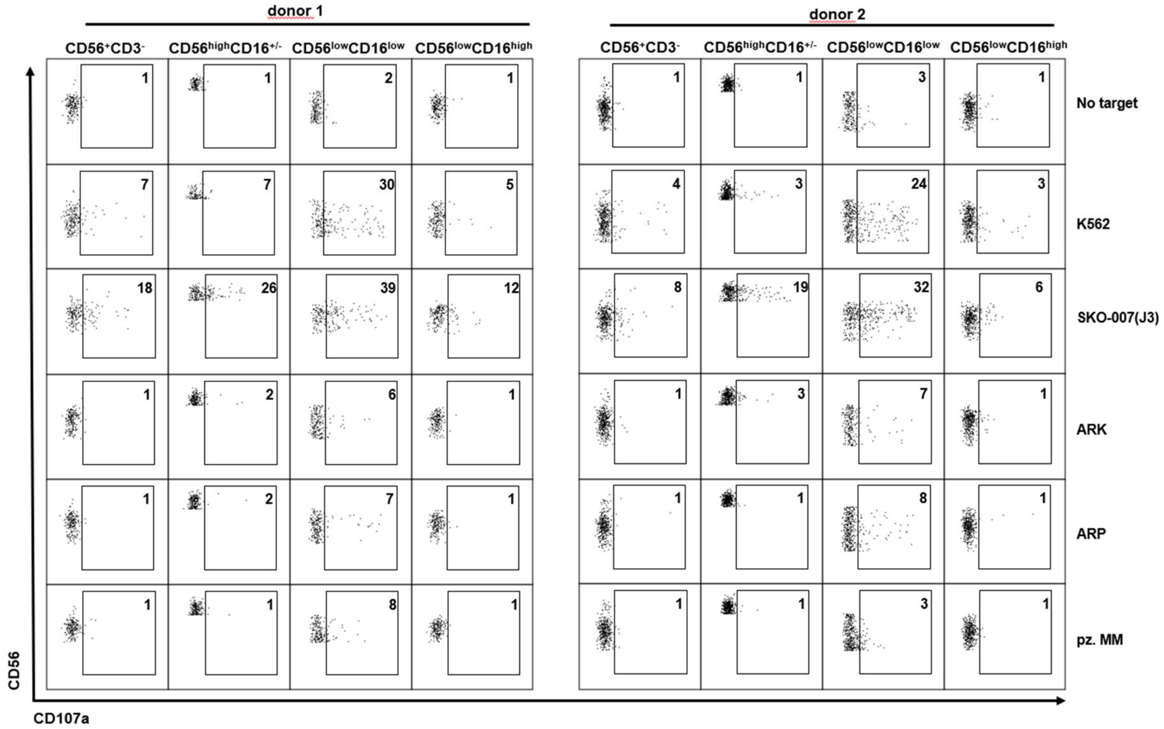

Figure S1. PB derived cells from two representative healthy donors were incubated for two hours without target or with distinct target cells including K562 and various MM cell lines (SKO-007(J3), ARK, ARP) and primary plasma cells (pz MM) as indicated. Cells were harvested and stained with anti-CD3, anti-CD56, anti-CD16 and anti-CD107 antibodies. NK cell subsets were analyzed considering the expression levels of CD56 and CD16. The percentage of CD107^{+} cells is shown in each plot.

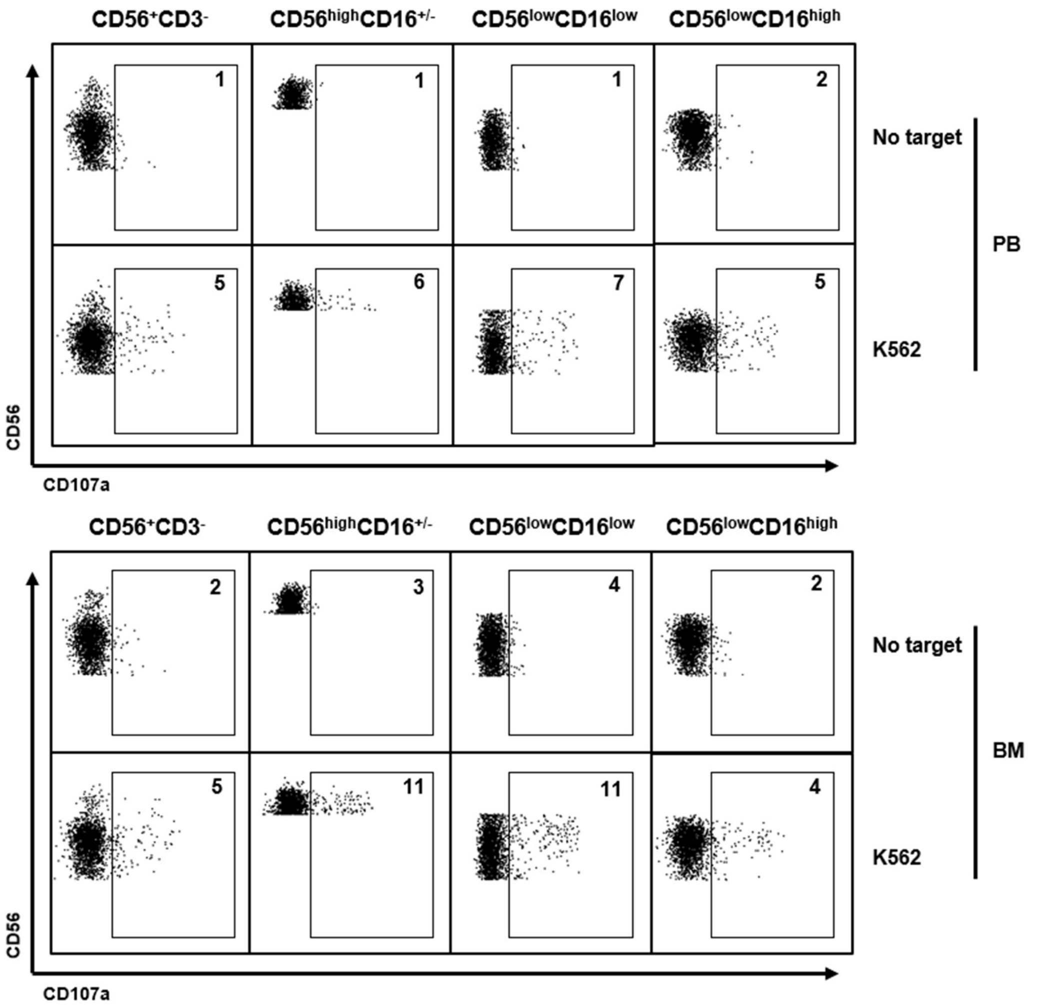

Figure S2. PB and BM derived cells from the same representative patient were incubated for two hours with or without the target cell line, K562. Cells were harvested and stained with anti-CD3, anti-CD56, anti-CD16 and anti-CD107 antibodies. Total NK cells and NK cell subsets were analyzed considering the expression levels of CD56 and CD16. The percentage of CD107$^+$ cells is shown in each plot.

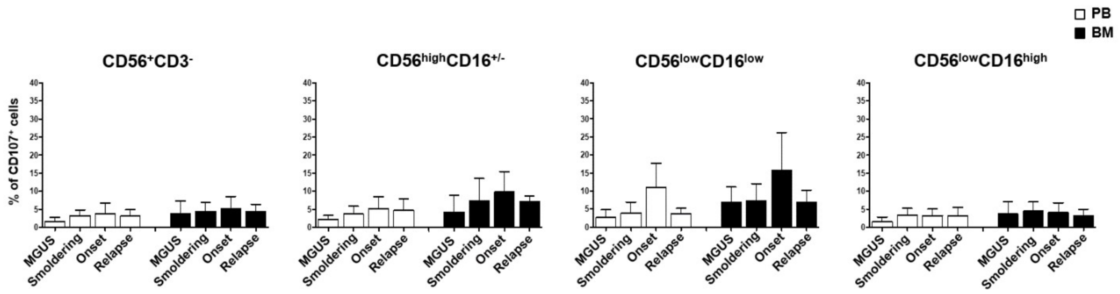

Figure S3. PB and BM derived cells were incubated for two hours with or without the target cell line, K562. Cells were harvested and stained with anti-CD3, anti-CD56, anti-CD16 and anti-CD107 antibodies. NK cell subsets were analyzed considering the expression levels of CD56 and CD16. The percentage of CD107$^+$ cells is shown and represents the net value of CD107 degranulation obtained as follow: (% of CD107$^+$ cells with target)-(% of CD107$^+$ cells without target). No significant differences were found between PB and BM NK cell degranulation in all the subsets analyzed. Patients are the same showed in figure 3 (MGUS, n = 6; Smoldering, n = 4; Onset, n = 5; Relapse, n = 4).

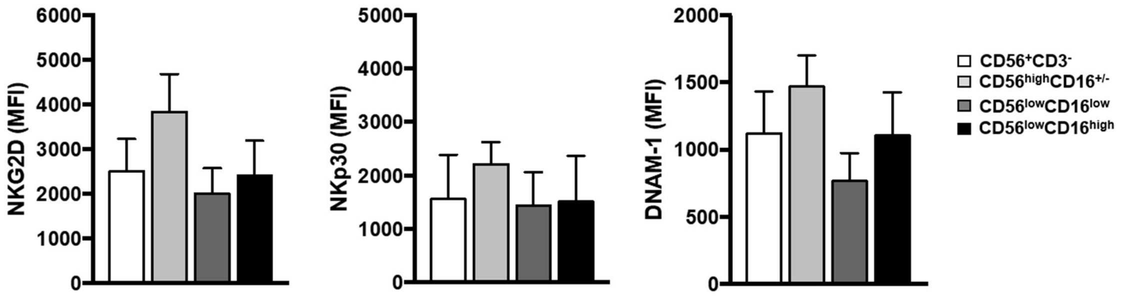

Figure S4. Expression levels of NK cell receptors on NK cell subsets derived from healthy PB donors. FACS analysis of surface expression of NKG2D, NKp30 and DNAM-1 on total NK cells and NK cell subsets in PB derived cells from 8 healthy donors. Values are expressed as mean of the MFI value and error bars represent SD.

Table S1. Therapeutic history of relapsed patients.

Patient	Chemotherapy First line	Second line	Third line	Number of relapse
**R1**	Lenalidomide<br>Dexamethasone	Melphalan<br>Prednisone<br>Lenalidomide		2
**R2**	Bortezomib<br>Prednisone			1
**R3**	Lenalidomide<br>Cyclophosphamide<br>Prednisone			1
**R4**	Bortezomib<br>Melphalan<br>Prednisone	Lenalidomide<br>Dexamethasone		2
**R5**	Adriablastin<br>Dexamethasone<br>Vincristine			1
**R6**	Adriablastin<br>Dexamethasone<br>Vincristine			1
**R8**	Adriablasin<br>Dexamethasone<br>Vincristine	Melphalan<br>Prednisone<br>Talidomide<br>Lenalidomide		2
**R9**	Bortezomib<br>Melphalan<br>Prednisone			1
**R10**	Adriablastin<br>Dexamethasone<br>Vincristine	Bortezomib	Lenalidomi<br>de	3
**R11**	Melphalan<br>Prednisone<br>Talidomide			1
**R12**	Velcade<br>Doxorubicin<br>Dexamethasone	Bortezomib<br>Melphalan<br>Prednisone	Velcade	3
**R13**	Adriablastin<br>Dexamethasone<br>Vincristine	Bortezomib		2
**R14**	Melphalan<br>Prednisone			1

	Talidomide			
**R16**	Velcade<br>Cyclosphosphamide	Velcade<br>Doxorubicin<br>Dexamethasone		2
**R17**	Adriablastin<br>Dexamethasone<br>Vincristine	Talidomide<br>Dexamethasone	Velcade<br>Dexametha<br>sone	3
**R18**	Lenalidomide			1

© 2018 by the authors. Licensee MDPI, Basel, Switzerland. This article is an open access article distributed under the terms and conditions of the Creative Commons Attribution (CC BY) license (http://creativecommons.org/licenses/by/4.0/).
